# A qualitative study of home- versus facility-based TB treatment for adolescents in Lima, Peru

**DOI:** 10.5588/pha.25.0025

**Published:** 2025-12-03

**Authors:** B. Roman-Sinche, F.M. Doradea, K. Tintaya, L. Lecca, S.S. Chiang

**Affiliations:** 1 Socios En Salud Sucursal Perú, Lima, Perú;; 2 A.R. Sanchez, Jr. School of Business, Texas A&M International University, Laredo, TX, USA;; 3 Department of Global Health and Social Medicine, Harvard Medical School, Boston, MA, USA;; 4 Unidad de Epidemiología Clínica, Facultad de Medicina, Universidad Peruana Cayetano Heredia, Lima, Perú;; 5 Department of Pediatrics, Warren Alpert Medical School of Brown University, Providence, RI, USA;; 6 Center for International Health Research and Division of Pediatric Infectious Diseases, Hasbro Children’s at Brown University Health, Providence, RI, USA.

**Keywords:** tuberculosis, digital adherence technologies, patient-centred care, focus groups

## Abstract

**BACKGROUND:**

Prior to the COVID-19 pandemic, TB treatment in Peru was delivered almost exclusively through facility-based directly observed therapy (DOT). During the pandemic, selected adolescents were allowed home-based TB treatment under caregiver supervision, along with video-supported treatment (VST) for adolescents with access to a smartphone.

**METHODS:**

We conducted 16 focus groups, each with 4–10 participants of adolescents with rifampicin-susceptible TB, their caregivers, and health care workers (HCWs). We used semi-structured guides to gather perspectives on facility-based DOT versus home-based treatment. Two authors applied inductive thematic analysis and identified emerging themes.

**RESULTS:**

HCWs assigned facility-based DOT or home-based treatment based on subjective assessment of the adolescent’s level of responsibility and support from caregivers. Almost all adolescents and caregivers preferred home-based treatment because it reduced disruptions to routine activities, TB-related stigma, and costs. A few disagreed, stating that facility-based DOT was better for guaranteeing adherence and support for adverse drug reactions. Most HCWs preferred facility-based DOT because they did not feel confident about adherence without direct visualisation by a provider, even with VST; moreover, they found VST to be time-consuming.

**CONCLUSION:**

Home-based TB treatment benefits adolescents and caregivers but must be further modified to achieve feasibility and acceptability among HCWs.

In 1994, to combat the surging TB epidemic by strengthening treatment adherence, the WHO endorsed directly observed therapy (DOT), or supervision by a trained individual of people taking TB medications.^1^ Since then, DOT has been considered a key component of TB treatment. Data from well-resourced settings show that DOT has curbed the incidence of TB and drug-resistant TB.^2^ In these settings, however, DOT is often patient-centred, that is, delivered in a way to support people with TB and accommodate their needs; for example, a health care worker (HCW) can bring the medication to the person at a convenient time and place and provide support for side effects or other concerns.^3^ In contrast, in high-burden settings, DOT often is health facility–based with limited hours, rendering it inconvenient.^3^ Recently, the WHO recommended that TB treatment be delivered in the patient’s community or home, rather than a health facility, because of higher rates of treatment success in these settings.^4^ Additionally, the WHO conditionally recommended video-supported therapy (VST; providing support and observing medication ingestion through video calls). None of the evidence cited by the WHO was adolescent-specific, and questions remain regarding treatment delivery for this population.

Before the COVID-19 pandemic, Peru’s Ministry of Health (*Ministerio de Salud*, or *MINSA*) required people to receive TB treatment via facility-based DOT, with exceptions for extenuating circumstances, such as a physical limitation that prevented someone from going to the health centre.^5^ In March 2020, in response to the COVID-19 pandemic, *MINSA* allowed HCWs to select adolescents for home-based TB treatment, with VST whenever possible, under the supervision of a responsible caregiver.^6^ We took this opportunity to evaluate the preferences of adolescents (10–19 years old) with rifampicin-susceptible TB disease, their primary adult caregivers, and HCWs with respect to home-based TB treatment versus facility-based DOT.

## METHODS

Peru has an estimated TB incidence of 173 per 100,000 population.^7^ The country’s TB epidemic is centred in the Lima-Callao metropolitan area, where this study took place in 2022.^8^ In Peru, 10%–12% of people who fall ill with TB are adolescents,^8^ and 74% of people with TB receive treatment at *MINSA*-operated community health centres,^9^ which have separate, marked entrances to TB treatment areas. People are assigned to the health centre closest to their home.

### Participants and sample size

We enrolled adolescents who initiated treatment for rifampicin-susceptible TB treatment between September 2020 and February 2021; their primary adult caregivers; and nurses and nurse technicians (the equivalent of licensed vocational nurses) who had worked in TB care at community health centres for ≥6 months. Adolescents were identified from a list of participants of a prior study who gave permission to be invited to participate in future studies.^10^ We purposively sampled adolescents who, based on prior interactions with study personnel, were thought to be more likely to share their experiences and opinions. We invited adolescent and caregiver participants from the northern, eastern, and central areas of Lima.

We invited each adolescent’s primary adult caregiver during TB treatment to participate. However, not all adolescents had an adult caregiver who participated. In some cases, caregivers declined to participate, citing time constraints; in others, adolescents did not live with caregivers. We also invited a convenience sample of nurses and nurse technicians to participate; not all HCWs worked in health centres where participating adolescents received treatment. We did not pre-specify a sample size but rather conducted focus groups until we achieved data saturation, that is, the point at which new data become redundant of already collected data,^11^ for each participant type (adolescents, caregivers, or HCWs).

### Data collection

Authors BRS and KT moderated focus groups in Spanish using semi-structured guides. Focus groups lasted 40–60 min each and took place in a private room at *Socios En Salud Sucursal Perú*. With participants’ consent, moderators took notes and audio-recorded the sessions. Recordings were transcribed verbatim.

### Analysis

Authors BRS and FD independently carried out each step of inductive thematic analysis, which summarises textual data by identifying recurring ideas, perspectives, and patterns across focus groups. In the first step, they read and listened to the transcripts to become familiar with the content. Second, they derived an initial codebook to capture participant experiences and perceptions. The codes were piloted on a few transcripts and revised until a final codebook was sufficient for capturing all relevant content. During the piloting step, BRS and FD strengthened inter-rater reliability by assessing the consistency of code application and resolving differences. Then, they applied the final codebook to all transcripts and identified emerging themes. Excel (Microsoft, Redmond, WA, USA) was used to organise transcript excerpts under each code. After each step, BRS and FD compared results and resolved differences through consensus, with SSC arbitrating disagreements as needed.

### Ethical statement

This study was approved by the institutional review board of Peru’s National Institute of Health (protocol #OEE-001-18). Participants ≥18 years old provided written informed consent. Participants 10–17 years old gave informed assent in addition to the written consent of their parent or legal guardian.

## RESULTS

We conducted 16 focus groups: 7 of adolescents, 6 of caregivers, and 3 of HCWs. Each focus group comprised 4–10 participants. In total, 47 adolescents, 41 caregivers, and 19 HCWs participated. Table 1 details the adolescents’ demographic and treatment characteristics. Of caregivers, 37 (90.2%) were female. HCWs worked a median of 5 (interquartile range: 4, 7) years in TB care; all were female. We conducted fewer HCW focus groups because data saturation was achieved more quickly in this participant group. All supporting quotes can be found in Tables 2–6.

**TABLE 1.** Characteristics of adolescent participants (N = 47).

	Median (IQR)
Age (years)	17 (1.8)
	Frequency (%)
Female gender	22 (47%)
Parent or guardian participated in the study	41 (87%)
Type of treatment received	
Facility-based DOT	19 (40%)
Home-based treatment, no VST	20 (43%)
Home-based treatment with VST	8 (17%)

DOT = directly observed therapy; IQR = interquartile range; VST = video-supported therapy.

**TABLE 2.** Supporting quotes related to criteria for home-based treatment: patient selection and procedures.

Theme	Supporting quotes
Criteria for selecting adolescents for home-based treatment	‘I am supportive of VST, but for patients who are committed and responsible with taking treatment.’ – Health care worker group 1
	‘Patients who were responsible were given (the medication to take at home). Patients who did not comply with video calls, we stopped giving them (the medication); they came in person. The issue of criteria, we also consider the vulnerability, the risk factor of the person, (…) and HIV risk factor.’ – Health care worker group 1
Procedures for home-based treatment	‘I had to take the package of the pills with me (…) the doctor told me that, as soon as I finished what they gave me each week, I had to go and pick them up.’ – Adolescent group 1
	‘My daughter was not supervised (…) It was up to me whether I gave her the pills or not (…) I did give her all her pills daily or every other day, as (her dosage) after a while went down until she finished her treatment.’ – Caregiver group 5

**TABLE 3.** Quotes illustrating adolescent and caregiver preference for home-based treatment.

Subtheme	Supporting quotes
Cost of transportation	‘It is also the cost because there are (patients) who are far away, such as in my case (…) I also missed (appointments) because I had to take a car and a motorcycle (…) it was far away for me, and to mobilise and all that daily.’ – Adolescent group 7
	‘Sometimes the transportation, in my case (…) I go down (to the health centre) for two soles and to come back up they charge me four soles (…) You can imagine, going down (to the health centre) every day. How would I cover the expenses? Going down, I could go down walking, but to come back up, the motor taxi charges me four soles at the stop.’ – Caregiver group 4
Less disruption of adolescent routines	‘It’s good to send the video because you feel like you’re not taking the treatment, it’s only a couple of minutes, you take your pills, and you go on with your normal life.’ – Adolescent group 6
	‘I feel it was good to send videos, yes, since I had to study, eh (…) I had to have breakfast, I would send the videos and I would begin to study (again), because, if I went to the health centre, my schedules conflict (…) So sending videos was the most viable for me.’ – Adolescent group 2
No conflict with caregiver work schedule	‘In my case, it is also the same. My daughter studies, I started working (…) and she may suddenly lie to me and not go (to the health centre). I prefer to receive (home-based treatment) so I can supervise her for a while.’ – Caregiver group 4
Less stigma	‘Of course, in this regard, I also think it would be better with video calls, no? (The patients should) be given their pills every 2 or 3 days. Because many of the kids, when they go to a health centre, they often run into friends there, and then their friends start spreading rumours, like, “Look! They have TB. Don’t get close to them. Be careful,” and then there’s one rumour here, another rumour there, you know?’ – Caregiver group 1
Less exposure to COVID-19	Participant 1: (Home-based treatment) seems good to me because, as there was a pandemic and everything, you could take the treatment and not go out too much, (not go) every day to take your pills at the health post.
	Participant 2: Aha, to infect to others. – Adolescent group 2

**TABLE 4.** Quotes illustrating adolescent and caregiver preference for facility-based directly observed therapy.

Subtheme	Supporting quotes
Better supervision of adherence and support for side effects	‘To take it at the health centre, because sometimes we don’t know if the family member is really watching us take the pills. We might also just keep it in our mouth – we don’t know, what if we are not taking it and then are not able to heal, right? Or if we do take it, we could also have side effects.’ – Adolescent group 6
	‘…With video calls, they can make a video and send it, sure, but how will the nurse know if they swallowed the pill or not? They can send a video pretending that they’re taking the medication, but, in reality, they could throw it away.’ – Caregiver group 3
	Participant 1: No, it is preferable to take it there, let (health providers) see for themselves if we have symptoms or not.
	Moderator: Uhum, ok, in your case which would you prefer?
	Participant 2: Go to the facility.
	Moderator: Also go to the facility, why would you like that?
	Participant 2: So that they can verify that we are taking the pills. – Adolescent group 6
Discomfort recording videos	‘Yes, truly, for me, in-person was better than having to record myself every day taking my pills (…) It’s uncomfortable having to take your pills while being recorded, I mean.’ – Adolescent group 3

**TABLE 5.** Quotes illustrating health care provider preference for facility-based directly observed therapy.

Subtheme	Supporting quotes
Distrust that adolescents are taking pills at home	Participant 1: For me, (home-based treatment) is also not 100% reliable.
	Participant 2: It’s not reliable.
	Participant 3: Because it’s one thing to see (the patient take it), right? (We can) through the video call, but it’s not the same as seeing them (in person), right?
	Participant 4: (Video calling) doesn’t assure you.
	Participant 5: Of course, of course, right?
	Participant 1: I don’t have that confidence that the patient is really taking the pills as they should, you know? – Health care worker group 2
Increased workload of home-based treatment	‘Having to call (patients) to tell them (…) to send their video, it’s a bit stressful, constantly calling. Whereas in person, they would come, and well, you’d just let them take the pills. I don’t see (home-based treatment) as beneficial because you’re continuously calling them to send (their video), to send it, and sometimes the messages or WhatsApp texts would come in the afternoon or at night. So, it’s a bit uncomfortable.’ – Health care worker group 1
	‘Sometimes there are patients who don’t take (their medication) on time. For example, you have to stay on top of calling them to send their video, say at 9:30, but they send it at 11 or 12, not on schedule. Or sometimes they record it and say, “I forgot to send the video,” and they send it in the afternoon or at night. But how do we really know if they took it in the morning or afternoon? So, (home-based treatment) is a benefit, and at the same time, well, the benefit is mainly for the patients. But in-person is the most important because we truly see that they are taking it.’ – Health care worker group 1
Lack of phone or data	‘Some (patients) still don’t have a cellphone with internet access, right? Or this app; so, in those cases, we relied on the support of a family member (to monitor treatment).’ – Health care worker group 1

**TABLE 6.** Quotes illustrating health care provider preference for home-based treatment.

Subtheme	Supporting quotes
Greater convenience for adolescents	‘I feel that forcing the patient to attend in person increases the likelihood of (treatment) abandonment, right? Because you’re forcing them to come down (to the clinic). It’s not a 1- or 2-month treatment – it’s 6 months, and if (they have) resistant (TB), it’s even worse, given their history. They get bored, they lose interest, and you have to find allies. In this case, who’s your ally? The family, right? And we have to let that young person – well, we already know that there’s a stage when they’re contagious and another when they’re not – and during that stage, I think we can be more flexible, as long as we’ve already assessed the patient’s and family’s commitment to the treatment (…) And the main thing is that the patient doesn’t get bored and can successfully complete the treatment.’ – Health care worker group 1
Decreased workload	‘… Yes, because (the work) decreased. In my case, my centre handles a large number of patients, and there is always activity in outpatient consultations. The (TB) patient flow decreased, and we were able to carry out other activities, such as improving the detection of symptomatic (TB) cases, which is ideal, right? Identifying and diagnosing early. And, well, mainly that, because my centre is also very small.’ – Health care worker group 1

### Criteria for selection of adolescents for home-based treatment

HCWs explained their criteria for selecting adolescents for home-based treatment. During the first 15 days of treatment, when the adolescent took medication at the health centre daily, HCWs observed whether the adolescent had support from an adult caregiver and subjectively assessed the adolescent’s commitment to adhere to treatment. Home-based treatment was offered to adolescents with caregiver support, who seemed committed to adhering to treatment, and who did not have frequent or severe adverse drug reactions (Table 2).

### Procedures for home-based treatment

For home-based treatment, adolescents or their caregiver made weekly trips to the health centre to pick up 1 week’s worth of medications. Whenever adolescents had access to a mobile phone, health providers used synchronous VST (i.e., WhatsApp video calls), asynchronous VST (i.e., adolescents sent them videos of themselves taking medication), or phone calls to confirm adherence. One adolescent was asked to send photos of himself taking pills (Table 2).

### Preference among most adolescents and caregivers for home-based treatment

Adolescents and caregivers had similar perspectives, with most preferring home-based treatment. Not all adolescents lived within walking distance of the health centre; for some, transportation costs were substantial. Home-based treatment was less disruptive to adolescents’ schooling, work, and other routines, which, in turn, allowed them to feel more ‘normal’ rather than like a sick person. In contrast, adolescents who received facility-based DOT had to ask for permission to miss work or school, as health centres were open only from 8 am to 2 pm. Some caregivers also preferred home-based treatment because they worked and could not accompany their children to facility-based DOT. In some cases, caregivers worried that adolescents would not actually go take their pills. They preferred to pick up the medication from the health centre and supervise adolescents taking treatment at home. Adolescents and caregivers appreciated that home-based treatment allowed them to avoid inadvertent disclosure of their TB status and its associated stigma if they were seen entering the TB treatment area of the health centre. Finally, many participants preferred home-based treatment because of their fear of contracting COVID-19 at the health centre (Table 3).

### Preference among a few adolescents and caregivers for facility-based DOT

Few adolescents and caregivers preferred facility-based DOT. These participants felt that facility-based DOT was better for ensuring adherence and addressing adverse reactions. Additionally, some adolescents preferred going to the health centre because they felt awkward recording videos of themselves taking medications (Table 4).

### Preference among most HCWs for facility-based DOT

Most HCWs preferred facility-based DOT. Despite acknowledging that facility-based treatment could be costly and time-consuming for adolescents and their families, HCWs identified two principal disadvantages of home-based treatment. First, HCWs did not trust that adolescents were adhering to treatment at home and stated that not even VST guaranteed medication adherence. Second, HCWs reported that adolescents often did not send videos of themselves taking medication or answer telephone or video calls as scheduled. Thus, HCWs spent a lot of time tracking down adolescents. Even when adolescents adhered to scheduled VST, HCWs found the process of calling adolescents to be too time intensive. For HCWs, facility-based DOT was more efficient because adolescents all arrived at the health centre within a short time window, and HCWs spent only a few minutes supervising each of them taking treatment. HCWs also reported that some patients did not have access to a mobile phone and/or data for VST or phone calls. In these cases, HCWs had to rely on caregivers to supervise adolescents’ adherence, but they felt uncomfortable not having direct confirmation of treatment administration (Table 5).

### Preference among a few HCWs for home-based treatment

A few HCWs preferred home-based treatment, even though they indicated that in-person treatment was more reliable. However, they pointed out that for some adolescents, home-based treatment was more feasible, due to its flexibility to accommodate academic or work schedules. HCWs working in small health centres liked that home-based treatment reduced the number of patients in the health centre needing attention at the same time. As a result, small health centres were not overcrowded. A couple of HCWs thought that home-based treatment was less burdensome for them, disagreeing with the vast majority. These HCWs felt that most of the adolescents under their care had responsible caregivers; thus, they did not have to spend time tracking down their videos and trusted that the adolescents were taking their medication (Table 6).

## DISCUSSION

Most HCWs in our study opposed home-based TB treatment for adolescents, citing increased workload for them and distrust that adolescents were adhering to medications, even with VST. However, a few HCWs dissented, acknowledging the benefits of home-based treatment for adolescents and their families. Most adolescents and caregivers preferred home-based treatment, which allowed them to continue daily routines without interruption and minimised the risk of inadvertently disclosing their TB status. These perspectives were consistent with our previous finding that facility-based DOT not only was less accessible for adolescents but also negatively impacted adherence (because of its inflexibility to accommodate schedule conflicts and travel) and household finances (due to transportation costs and the need for adult caregivers to miss work to accompany adolescents to the health centre).^12,13^ Adolescents in other countries receiving facility-based DOT reported similar difficulties.^14,15^ An international panel of experts, including adolescent TB survivors and youth advocates, endorsed community- (a location close to the person’s home or workplace) or home-based TB treatment for adolescents.^16,17^ Similarly, the WHO, based on evidence of better treatment outcomes, recommended community- or home-based TB treatment (for people of all ages) in lieu of facility-based DOT.^4^ Nonetheless, some adolescents preferred facility-based DOT because they felt this modality provided them with greater support during TB treatment. This preference should be respected, though ideally health centres should offer more flexible hours and take steps to ensure adolescents’ privacy, such as not forcing them to enter the health centre through a separate entranced marked for people with TB.

A striking observation from our study was the emphasis placed by participants, especially HCWs, on DOT as a form of supervision rather than support. The most common objection to home-based treatment, voiced by not only HCWs but also a few adolescents themselves, was lower reliability for ensuring adherence. This perspective is problematic because it ignores the challenges – including schedule conflicts between health centre hours and school, the large pill burden, long treatment duration, and adverse treatment events^12^ – that keep people with TB from taking treatment as prescribed. A conceptual reorientation of treatment from being ‘supervised’ to ‘supported’ may increase recognition of these challenges.^4^

Several strategies can be incorporated into community- or home-based treatment to enhance support for adolescents. One approach is peer support, which consists of accompaniment, treatment delivery, and/or health education by other adolescents or young adults via group and/or individual sessions. This approach also may increase the feasibility and acceptability of home-based treatment from the perspective of HCWs, who voiced strong concerns about adherence and increased workload. Neither the feasibility/acceptability nor the efficacy of peer support for improving treatment outcomes has been evaluated for adolescents with TB. However, a qualitative study from Russia, where children and adolescents are hospitalised for TB treatment, illustrated the positive impact of peer support for hospitalised adolescents in Tomsk.^18^ These adolescents formed strong friendships, which provided a respite from the stigma they experienced outside the hospital and enhanced their mental wellbeing. Among adolescents living with HIV, peer support led to improved treatment adherence and retention in care in some settings but not in others.^19^

Digital adherence technologies (DATs), such as phone-based technologies and digital pillboxes, can help remind people to take their pills. Various forms of DATs are less labour-intensive for HCWs than VST and may be more acceptable to adolescents who feel awkward using VST. Examples include 99DOTS, a strategy that involves people calling a different phone number each day to register pill ingestion.^20^ However, studies in diverse settings have demonstrated variable performance of DATs for supporting adherence.^21^

eHealth tools with gamification have been used to engage adolescents and youth in chronic disease management; this approach has not yet been used for adolescents with TB.^22^ Games commonly incentivise treatment adherence through rewards, virtual peer competitions, and interactive treatment progress displays.^23^ Several games use narratives and characters to teach adolescents how to manage their illness. Studies have not consistently demonstrated efficacy for adolescents’ self-management, but the evidence is limited by low study number and quality.^23^

The mixed findings on the efficacy of peer support, DATs, and eHealth tools with gamification may suggest that different treatment support strategies work for different individuals. Ideally, different options should be available, and each adolescent should receive a package of interventions individualised to their needs and preferences.^4^

This study had limitations. First, it was conducted during the COVID-19 pandemic. Although one reason why some participants preferred home-based treatment was to avoid contracting COVID-19, most of the findings were not specific to pandemic conditions. Focus group moderators selected adolescent participants based on their perception of who was more likely to share their perspectives. Not all caregivers were able to participate due to time constraints. Therefore, we may not have included the perspectives of participants with the most hardships.

## CONCLUSION

This study provides insight into the perspectives of key stakeholders on home- versus facility-based TB treatment for adolescents in Lima. Post-pandemic, *MINSA* continues to offer the option of home- or community-based treatment, especially for people with difficulties accessing the health centre for facility-based DOT.^24^ However, the frequency with which HCWs select home- or community-based treatment is unknown. Improving TB care for adolescents will require making home-based treatment more supportive for adolescents and less labour-intensive for HCWs.
